# Train the trainer: improving health education for children and adolescents in Eswatini

**DOI:** 10.4314/ahs.v22i1.76

**Published:** 2022-03

**Authors:** E McAdams, B Tingey, D Ose

**Affiliations:** 1 University of Utah, Department of Family and Preventive Medicine

**Keywords:** Health Education for Children, Adolescents in Eswatini

## Abstract

**Background:**

Eswatini has the highest HIV prevalence in the world. One issue at the root of health in Eswatini is a lack of basic health knowledge among children and adolescents, which amplifies the likelihood of disease transmission and poor health outcomes.

**Objectives:**

To address the lack of basic health education and to improve health knowledge, we developed and evaluated a medical education program to train shepherds (train-the-trainer) who are supporting children and adolescents at local CarePoints.

**Methods:**

To determine the change between pre-test scores and post-test scores, both a paired t-test and mixed-effects regression were performed.

**Results:**

The program had 67 total participants, mostly female (67.2%), with an average age of 27.1 years. Following the medical education program, participants had a statistically significant increase in post-training scores. Years of prior schooling, age, and gender did not show a statistically significant effect on post-training scores.

**Conclusion:**

Teaching basic health education knowledge to CarePoint shepherds is effective to increase their knowledge and awareness with respect to relevant health topics. This model of directed medical education could be expanded to other community members in Eswatini to bridge gaps in health knowledge and disease awareness. A similar model could be employed in other developing countries with limited health education and limited access to health information.

## Background

Eswatini (formerly known as Swaziland) is a small landlocked country bordered on three sides by South Africa and by Mozambique on one side. It is a developing country of approximately 1.3 million people (1), with an average life expectancy of 58 years (2), compared to 78.6 years in the US (3). This is in large part due to the highest HIV prevalence in the world of 27.3% as well as a high rate of tuberculosis (2). Both physician access and skilled health professional (4) availability is significantly lower in Eswatini as compared to the US (1.7 to 24.5 per 10,000 people, and 17.7 per 122.7 people, respectively). Childhood mortality under the age of 5 and adolescent birth rates are much higher in Eswatini, as is mortality from unsafe wash services (see Table 1) (4).

**Table 1:** Statistics comparing Eswatini health measures to those in the in United States

Incidence	Eswatini	United States
Life Expectancy at Birth (years)	57.7	78.5
Physician Access (per 10,000)*	1.7	24.5
Skilled Health Professionals (per 10,000)**	17.7	122.7
Childhood Mortality (under age 5, per 1000 live births)	70.4	6.5
New HIV Infections (per 1000)	9.37	1.74***
Adolescent Birth Rate (per 1000 ages 15 to 19)	87	22.3
Mortality from Unsafe Wash Services (per 100,000)	27.9	0.2

^#^All data taken from WHO's “World Health Statistics 2018: Monitoring health for the SDGs” (17), with exception of:

* World Health Statistics 2015 Report (1)

** World Health Statistics 2016: Monitoring health for the SDGs (4)

*** CDC HIV Surveillance Report 2017 (18)

Besides other contributing factors (such as access to healthcare, low household income), low health literacy is one important factor impacting poor health outcomes in Eswatini. Reasons for low health literacy includes many factors, such as decreased opportunity for basic education as evidenced by low secondary school enrolment rates (formal schooling after grade 7 enrolment ranges from 32.3% (male) to 37.7 % (female) (5). To address the lack of basic health education among the shepherds, we developed a medical education program covering a range of health topics (e.g., HIV). This study aimed to evaluate the effectiveness of this intervention.

## Methods

Adventures in Missions (AIM) is an international organization that has had a presence in Eswatini since the early 2000s. AIM initially started with a focus on feeding orphans across the country, using community resources already in place to assist. Over time, AIM has expanded to feed more than 8,000 children (many of whom are orphans) daily, as well as offer many other programs with an emphasis on sustainability and developing future country leaders. The AIM structure in Eswatini is such that these 8,000 children attend plots of land, called CarePoints, with local women who cook for the children daily. AIM employs ∼65 young adult men and women to serve in a leadership role as “shepherds” at each CarePoint. Shepherd's guide and oversee the daily activities of the children when they are not in school and serve as mentors and sometimes parental role models for the children. Most of the shepherds grew up in the CarePoint system and have been chosen as leaders among their peers. They have completed basic education through high school and speak English as a second language. Over the last decade plus, AIM has been working alongside community members and children at CarePoints around the country to implement garden growing projects, clean water projects, distribution of food containers, assistance with education through school fees, and leadership development opportunities (6).

### Aims of the Medical Education Program

The lack of healthcare education was addressed by developing and implementing a medical education program for the AIM staff and shepherds (with mandated attendance) at the AIM staff building in Manzini, Eswatini. The goal of the program was to increase the medical knowledge and health awareness of the shepherds, so that they can be empowered to serve as health advocates for children at CarePoints around the country, as well as health advocates for themselves and their local communities. The program is based on the so-called train-the trainer (TTT) concept. TTT is an educational model where potential trainers with strong ties to the community are targeted for training. These “trainers” (shepherds) are provided with education that enable them to, in turn, provide specific training or oversight to target audiences (children and adolescents). TTT is a widely acknowledged educational model across a number of disciplines, including public health (7).

### Development and Implementation of the Medical Education Program

Prior to the medical program, the shepherds and AIM staff were asked about medical topics that they desired to learn about, and this list of topics was communicated to author EM by AIM staff in advance of the medical education program. The list of topics provided by the shepherds were the basis for the large-group, small-group, and hands on training that was provided, such that it was a tailored educational approach to specifically address participants needs. The topics addressed are listed in Table 1 (with further details below) and included: puberty, sexually transmitted infections (STIs) including HIV, skin infections, diarrhoea, dehydration, personal and hand hygiene, abuse and domestic violence, wound care, common musculoskeletal injuries, common types of cancer and possible preventive measures, and asthma and signs of respiratory distress (8).

The program was 6 hours of training on day one and 7 hours of training on day two (including time for a lunch break on both days). Multiple methods of education were utilized, including large group sessions (67 participants in one location), small group sessions (∼15 persons per group), and hands-on training (∼ 15 persons per group). Where able, hands-on training and small group education were provided to create more interaction to maximize learning. The educational program included multiple break points at which questions could be asked covering any aforementioned or other health topic participants had. A colour-printed packet of all information taught was provided to each participant to allow for notetaking during informational sessions.

### Evaluation of the Program

Pre- and post-education tests covering the program topics were given to the participants, to assess impact of the education. The same questions were asked on both the pre- and post-tests; tests were designed by the author (EM) to ask questions about each different type of topic covered throughout the health education program. Test questions were formatted in a multiple-choice style, some of which had true/false answers, and other questions were “choose all that apply” answer fields. Each participant voluntarily signed a written consent to have their de-identified demographic information and test results shared through publication. Participants who agreed to have results shared were asked to provide information about how many years of formal education they had received in their lifetime and were permitted to interpret that however they wanted.

### Statistical analysis

To determine this change between pre-test scores and post-test scores, both a paired t-test and mixed-effects regression were performed. The paired t-test only allows analysis for individuals that have scores for both tests, so individuals with missing scores are removed. The mixed-effects regression uses all the data provided. The outcomes of these two tests are similar for the data analysed, suggesting that the results can be more confidently accepted, and selection bias is not a concern. Additionally, to assess if any of the recorded variables (years of schooling, age, and gender) in addition to pre/post-test status significantly impacted the change in scores, we applied also mixed-effects regression.

## Results

**Figure 1:**
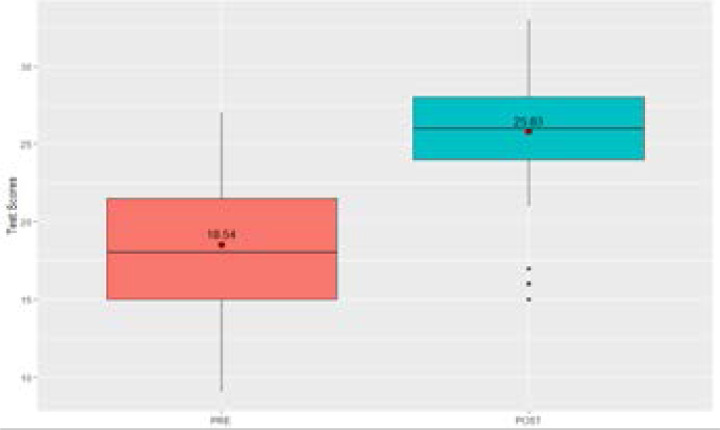
Comparison of Pre-Test Scores and Post-Test Scores

There were 67 total participants in the medical education program. Participants were pre-dominantly female (67.2%; n=45) with an average age of 27.1 (SD=6.2) years. All participants spoke English (most as a second language). Average amount of reported formal education was 13.4 years (SD=3.0) (Table 3). As seen in Figure 1, out of a total of 33 correct answers, participants scored an average of 18.5 (SD=4.5) correct answers on the pre-education test, and post-education test scores increased to an average of 25.8. While years of schooling (95% CI, -0.1, 0.5), age (95% CI, -0.2, 0.1), and gender (95% CI, -2.5, 1.3) did not show statistically significant impact on post-education test scores, completing the medical education training did show a statistical significance on posttest scores. Completing the medical education training was associated with an increase in baseline test score by an average of 7.1 across the participants (95% CI, 6.0, 8.2) (Table 4).

**Table 3:** Demographics of Participants in the Medical Education Program

Variables	Results
**n**	67
**Age Mean (SD)**	27.1 (6.2)
**Gender n (%)**	
Female	45 (67.2)
Male	22 (32.8)
**Years of Schooling Mean (SD)**	**13.4 (3.0)**
**Pre-Education Test Scores Mean (SD)**	**18.5 (4.5)**

**Table 4:** Factors Impacting Test Scores*

	Overall Change in Score from Baseline	95% Confidence Interval	Statistical Significance
**Training (Post)**	7.1	(6.0, 8.2)	***
**Years of Schooling**	0.2	(-0.1, 0.5)	
**Age**	-0.1	(-0.2, 0.1)	
**Gender (Male)**	-0.6	(-2.5, 1.3)	

Only training had a significant impact on change in test scores. Changing from pre-scores to post-scores had an average test score increase of 7.1 across the participants.

## Discussion

The aim of this study was to evaluate a health education program for CarePoint shepherds. The results of our evaluation have shown that this two-day health education conference can be effective in improving the health knowledge of the participants. And most notably, the improvement in pre- and post-education scores did not depend upon gender, age, or prior years of schooling, but solely on participating in the two-day conference.

### Comparison with other literature

This health education program is based on the TTT concept, where shepherds (trainer) are trained to enable them, in turn, to provide health education to children and adolescents in CarePoints. To the best of our knowledge, our evaluation is the first publication describing the implementation of this approach in Eswatini. However, overall, TTT is a well-established and often used concept in approaches aiming to strengthen global health^9^. For example, the study of Tartari et al. (2019) has shown that TTT can be effective in enhancing participant's knowledge about hand hygiene (10). Also, as shown with the study of Hiner et al. (2009), TTT can be an effective and sustainable method in HIV counselling^11^.

### Implications for practice and further development

While universal or regular access to broadband internet is widely available in industrialized countries, this access is quite limited in developing countries; ultimately, this limits the ability to gain information about personal health, medical conditions, and diseases in developing countries^12^. In Eswatini, this holds true as well, and in an effort to improve baseline health literacy among young adult community leaders a multi-day health education conference was held.

The improved health knowledge (on topics addressed) over this two-day conference demonstrates that directed health education can improve baseline health knowledge in young adults in Eswatini.

Given that this education was well-received, we suggest that health education directed at adult community members in a developing country over a short amount of time can be effective at decreasing knowledge gaps about basic health education, with the ultimate hope that improved education could lead to future decrease in disease and improve actual health of communities.

This model of education could be adapted and shared in different formats with adults and children of rural communities in Eswatini who may not access the CarePoint or local health systems. Additionally, the concept of focused health education about common medical conditions and public health concerns, addressed to non-medically trained adults, could be expanded, and utilized in other countries to spread health knowledge with aims to improve health outcomes.

This is significant because it would provide a means for health education in areas that otherwise may lack direct access to health education through reliable internet (a concern in many developing countries). However, implementation of a similar educational program in any country would require an established relationship with the community members who would be participating and learning to ensure their interest in the program from the outset, as well as their input on what health education topics need to be taught.

### Strengths and limitations of the approach

Strengths of this education approach include teaching topics that were selected in advance by conference attendees to ensure both a desire to learn about the topics was present as well as a baseline knowledge deficit in these areas, rather than make any assumptions about areas of knowledge gaps. Another strength of this educational program includes the style of teaching utilized, which included both large and small group settings, with hands-on demonstration and interactive discussions when possible (13–15) to better engage the target audience.

One weakness of the educational program includes that results from pre- and post-education training could be skewed to appear more favorable given that the baseline knowledge of topics covered was low (as suggested by the group's desire to be educated about the topics covered). Another possible limitation of this intervention is that the pre- and post-education training tests had the same questions to assess for change in knowledge over material taught. This method of assessing for learning or improved baseline knowledge could have primed the participant to focus on only specific information from the teachings as targeted from the test questions rather than learn more broadly about the topics that were covered (16). However, despite this possibility, it is notable that as demonstrated in our results, the only statistically significant impact on post-education scores was having received the training (not age, gender, or years of schooling prior to the health education training).

## Conclusion

Gaps in education are a root cause of both poverty and poor health statistics and outcomes. In order to see a decrease in mortality rates, a drop in communicable disease transmission, and an increase in life expectancy (in any country), there must be a focus on education. Having the knowledge of what causes disease as well as an understanding of how disease can spread or worsen, and an understanding of preventive measures to minimize certain conditions, can provide individuals and communities the power to make decisions that impact the health of their country.

As shown with our study, teaching basic health education knowledge to CarePoint shepherds can be effective to increase their knowledge and awareness with respect to relevant health topics. This model of directed medical education could be expanded to other community members in Eswatini to bridge gaps in health knowledge and disease awareness. A similar model could be employed in other developing countries with limited health education and limited access to health information.

## Figures and Tables

**Table 2:** Topics taught in the medical education program

Topic Discussed	Description	Relevance
Puberty	Process of normal puberty in boys and girls, including debunking myths related to this process	Public education about normal development and puberty is lacking in Eswatini
HIV	Immune system impact, transmission, signs, symptoms, preventive and treatment options	Eswatini has highest HIV prevalence in the world; life expectancy is 57 years vs. 78 years in developed countries
Other Sexually Transmitted Infections (STIs)	Education of signs, symptoms, treatment for e.g. chlamydia, gonorrhoea, syphilis, or HPV	Those at risk for HIV are at risk for other STIs
Skin Infections	Examples of common bacterial, viral, and fungal conditions (and those at higher risk in HIV positive patients). Discussion of who needs to be seen by a healthcare provider	Skin infections spread more easily in areas with little healthcare access or education; certain skin infections are signs of life-threatening illnesses
Diarrhoea	Causes, discussion of who should be seen by healthcare provider	Diarrheal illness is common in the developing world.
Dehydration	Signs and symptoms. Treatment with rehydration to prevent water intoxication and complications	The life-threatening consequence of diarrhoea, that is most often preventable
Personal Hygiene	Proper hand, teeth, body, hair hygiene to minimize disease spread	Proper hygiene minimizes the risk of spreading communicable disease
Recognizing Signs of Abuse and Domestic Violence	Signs of abuse, local resources for suspected abuse, small group conversations about abuse cases	Abuse is common around the world. Knowing how to recognize this and what to do for those affected can be lifesaving.
Wound Care	Review of materials in CarePoint first aid kits with hands-on practice	Each CarePoint has a first aid kit, but shepherds had no prior training about how to use items in the kit
Musculoskeletal Injuries	Education about how to properly wrap sprained ankles, knees, and wrists including hands on practice	MSK injuries are common among children in rural Eswatini; appropriate support for minor injuries can improve comfort and expedite healing
Asthma	What it is, triggers and treatment. Demonstrations of signs of respiratory distress.	Asthma is a common condition. Knowing signs of respiratory distress and learning proper inhaler use can save lives.
Cancer	Review of top 10 causes of cancer; signs, symptoms, treatment, and any preventive options	Minimal education is provided about common cancers in Eswatini, some of which have preventable measures
